# Three-Dimensional Bioprinting of GelMA Hydrogels with Culture Medium: Balancing Printability, Rheology and Cell Viability for Tissue Regeneration

**DOI:** 10.3390/polym16101437

**Published:** 2024-05-19

**Authors:** Laura Mendoza-Cerezo, Jesús M. Rodríguez-Rego, Antonio Macías-García, Antuca Callejas-Marín, Luís Sánchez-Guardado, Alfonso C. Marcos-Romero

**Affiliations:** 1 Department of Graphic Expression, School of Industrial Engineering, University of Extremadura, Avenida de Elvas, s/n, 06006 Badajoz, Spain; lmencer@unex.es (L.M.-C.); acmarcos@unex.es (A.C.M.-R.); 2 Department of Mechanical, Energy and Materials Engineering, School of Industrial Engineering, University of Extremadura, Avenida de Elvas, s/n, 06006 Badajoz, Spain; amacgar@unex.es; 3 Department of Anatomy, Cell Biology and Zoology, Faculty of Science, University of Extremadura, Avenida de Elvas, s/n, 06006 Badajoz, Spain; acallejas@unex.es (A.C.-M.); guardado@unex.es (L.S.-G.)

**Keywords:** biomaterials, bioprinting, GelMA, rheology, printability

## Abstract

Three-dimensional extrusion bioprinting technology aims to become a fundamental tool for tissue regeneration using cell-loaded hydrogels. These biomaterials must have highly specific mechanical and biological properties that allow them to generate biosimilar structures by successive layering of material while maintaining cell viability. The rheological properties of hydrogels used as bioinks are critical to their printability. Correct printability of hydrogels allows the replication of biomimetic structures, which are of great use in medicine, tissue engineering and other fields of study that require the three-dimensional replication of different tissues. When bioprinting cell-loaded hydrogels, a small amount of culture medium can be added to ensure adequate survival, which can modify the rheological properties of the hydrogels. GelMA is a hydrogel used in bioprinting, with very interesting properties and rheological parameters that have been studied and defined for its basic formulation. However, the changes that occur in its rheological parameters and therefore in its printability, when it is mixed with the culture medium necessary to house the cells inside, are unknown. Therefore, in this work, a comparative study of GelMA 100% and GelMA in the proportions 3:1 (GelMA 75%) and 1:1 (GelMA 50%) with culture medium was carried out to determine the printability of the gel (using a device of our own invention), its main rheological parameters and its toxicity after the addition of the medium and to observe whether significant differences in cell viability occur. This raises the possibility of its use in regenerative medicine using a 3D extrusion bioprinter.

## 1. Introduction

Tissue engineering is a specialised field of study in the application of life sciences together with materials engineering to achieve the restoration, maintenance and improvement of tissue function [1]. It can therefore be used to repair or regenerate various types of damaged tissue through the construction of suitable scaffolds that can mimic different tissue structures [2].

In recent years, 3D bioprinting has become increasingly important in the generation of three-dimensional scaffolds capable of mimicking different tissue structures [3]. It is a fast and inexpensive method for producing geometrically well-defined scaffolds [4], which also allows the incorporation of cells into their matrix, facilitating their precise placement. Bioprinted scaffolds can mimic the in vivo microenvironment to accurately model cell behaviour in vivo [5]. To study the complex cell–matrix interactions that drive cell differentiation and self-organisation in vitro, it is necessary to mimic the dynamic nature of the extracellular matrix (ECM) [6]. Some of the materials that can be used to generate bioprinting scaffolds are compounds of natural origin, such as collagen, gelatine, chitosan or alginate, among others, while others can be synthetic, such as PCL, PLA, PEG or Pluronic [7].

Hydrogels consist of a three-dimensional network of molecules composed of polymer chains with the capacity to absorb up to a thousand times their dry weight in a water-rich environment and are used as bioinks in bioprinting because of their structural similarity to the native ECM [8]. Such hydrogels are formed through the physical, ionic or chemical cross-linking of the polymer chains of which they are composed [9]. Of the different types of hydrogel bioinks currently used in bioprinting, those based on methacrylated gelatine (GelMA) are very promising due to their good biocompatibility, on-demand photoreticulation and widely tuneable physicochemical properties [10]. The ECM has a high content of collagen, which is the most abundant protein, so gelatine, a product of collagen hydrolysis, is a single-stranded biopolymer very similar to collagen, containing lysine groups and free amines that can be modified to form a processable biopolymer.

GelMA hydrogels are made by reacting gelatine and methacrylic anhydride (MA) in the presence of a photoinitiator [11], which can undergo free radical polymerisation (chemically cross-link when exposed to ultraviolet light) [12]. In addition, the processing temperature also plays an important role in the mechanical properties of GelMA, which undergoes physical gelation at a sol–gel transition temperature. When cooled below the gel point, it undergoes a conformational transition from a coiled polymer to a collagen-like helical structure, whereas when heated above the transition temperature it reverts to a solution [13].

Knowledge of the various rheological properties and printability variables of hydrogels allows their suitability for the bioprinting process to be determined by providing key information on the behaviour of the material throughout the printing steps [14]. Properties such as viscosity, storage modulus and yield strength are directly related to the printability of the materials, while shear recovery behaviour is related to the stability of the prints produced [15]. Thus, flow behaviour and consistency index relate to the ease with which materials are extruded through the bioprinter nozzle, yield strength to the minimum force required for the material structure to collapse and flow and viscosity to the ability of the hydrogel to adhere to the extruder walls and block the nozzle [16]. 

In terms of cell viability, the culture medium is essential to maintain the conditions necessary for cell survival and cell proliferation and differentiation. In this work, the rheological characterisation of the culture medium composed of DMEM (Dulbecco’s Modified Eagle Medium), FBS (foetal bovine serum) and antibiotics, suitable for HeLa cell culture [17], will be carried out in order to know how it will behave during bioprinting and to predict the possible changes it can cause in different hydrogels. 

Furthermore, knowing the rheological and printability properties that the hydrogel acquires after the incorporation of cells together with the culture medium and the aforementioned change that this produces in the internal structure of the hydrogel allows one not only to know the real behaviour of the hydrogel when bioprinted with cells but also to consider increasing the concentration of the culture medium to ensure greater stability of the cells and to reduce as little as possible the mechanical and printability properties of the hydrogel.

## 2. Materials and Methods

### 2.1. Starting Materials

The following starting materials were used for this work.

#### 2.1.1. GelMA Lyophilizate

We used hybrid hydrogel made from porcine gelatine with properties suitable for 3D bioprinting [18,19], thanks to its 50% functionalisation with methacrylate groups that induce cross-linking of the hydrogel when exposed to UV light, increasing the structural capacity of the hydrogel. 

GelMA Lyophilizate was supplied by CELLINK (Gothenburg, Sweden).

#### 2.1.2. Culture Medium and PBS

The culture medium used was DMEM (Dulbecco’s Modified Eagle Medium) with 10% FBS (foetal bovine serum) and 1% penicillin/streptomycin, suitable for HeLa cell culture. DMEM was supplied by Thermo Fisher (Waltham, MA, USA).

FBS was supplied by Thermo Fischer and the antibiotic (penicillin/streptomycin) by Sigma Aldrich (St. Louis, MO, USA).

PBS was supplied by Thermo Fisher.

#### 2.1.3. Reconstitution Agent P

Reconstitution agent P is a buffered solution that allows the freeze-dried state of the GelMA hydrogel to be reversed. Specifically, it is composed of phosphate-buffered saline and HEPES buffer solution, providing the hydrogel with the appropriate osmolarity and pH conditions. 

Reconstitution agent P was supplied by CELLINK.

#### 2.1.4. Sodium Hydroxide (NaOH) and Hydrochloric Acid (HCl)

Both NaOH and HCl were used to adjust the pH of the solution to 7.0–7.4, which is the appropriate range for achieving both correct hydrogel properties and cell viability.

NaOH and HCl were supplied by PanReac AppliChem (Chicago IL, USA). 

#### 2.1.5. Cells

The HeLa cell line (human cervical carcinoma) was purchased from the Research Support Services of the University of Extremadura (SAIUEx) and cultured in the culture medium described above (DMEM with 10% foetal bovine serum and 1% penicillin/streptomycin 1%), in a humidified air atmosphere of 5% CO_2_ at 37 °C [20].

### 2.2. Equipment

The following equipment was used to carry out this work:

- Biological binocular microscope AE-20, MOTIC (Barcelona, Spain).
- Epifluorescence optical microscope Nikon Eclipse 80i (Tokyo, Japan).
- Test device for the optimisation of 3D bioprinting, specially designed for the testing of different parameters defining the printability of hydrogels. Patent number EN 1 303 662 U.
- Eppendorf Galaxy 48R CO_2_ Incubator.
- Vertical laminar airflow cabinet Mini V/PCR.
- Cellink BIO X Bioprinter from CELLINK.

### 2.3. Method

**Figure 1:**
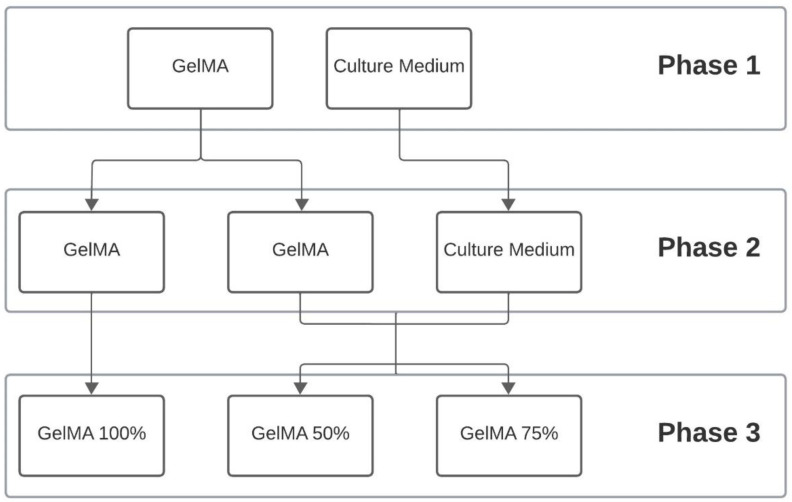
Work planning.

Figure 1 shows the outline of the development of the work divided by phases.

Phase 1 involved the characterisation of the starting materials. To this end, rheological analyses were carried out on the culture medium, while GelMA 10% was subjected to both rheological analyses and printability tests. 

To carry out the proposed study, phase 2 proposed the different mixtures that would form the final hydrogels (GelMA 100%, GelMA 75% and GelMA 50%). 

Finally, in phase 3, rheological analyses, printability tests and viability tests were carried out on the three hydrogels proposed in the study to compare the influence of the addition of culture medium on the properties of GelMA.

#### 2.3.1. Preparation of Hydrogels 

The concentration chosen for the reconstituted hydrogel was 10% (100 mg/mL), as this is the concentration used in most GelMA studies [21].

For reconstitution, photoinitiator was dissolved in reconstitution agent P at a concentration of 1 mg/mL.

The final solution was filtered through 0.22 µm syringe filters to remove any impurities or particles that might interfere with hydrogel cross-linking, as well as any bacteria, and added to the lyophilised GelMA vial. The mixture was heated at 50 °C for 60 min. Once the hydrogel was obtained, the pH was adjusted to 7.0–7.4 using NaOH or HCl.

#### 2.3.2. Proportion of Hydrogels

The ratio of the hydrogel (GelMA 10%) to the culture medium (DMEM) used in this study is shown in Table 1.

#### 2.3.3. Rheological Characterisation

The rheological properties of the hydrogels were determined at 37 °C (physiological temperature) using a NETZSCH Kinexus pro+ rotational rheometer model Discovery DHR-2, TA Instruments. A 20 mm parallel plate was used for the tests with a spacing between 450 and 550 µm, depending on the sample. The samples were allowed to stand for 20 min prior to testing to achieve mechanical and thermal equilibrium.

#### 2.3.4. Characterisation of Printability

**Figure 2:**
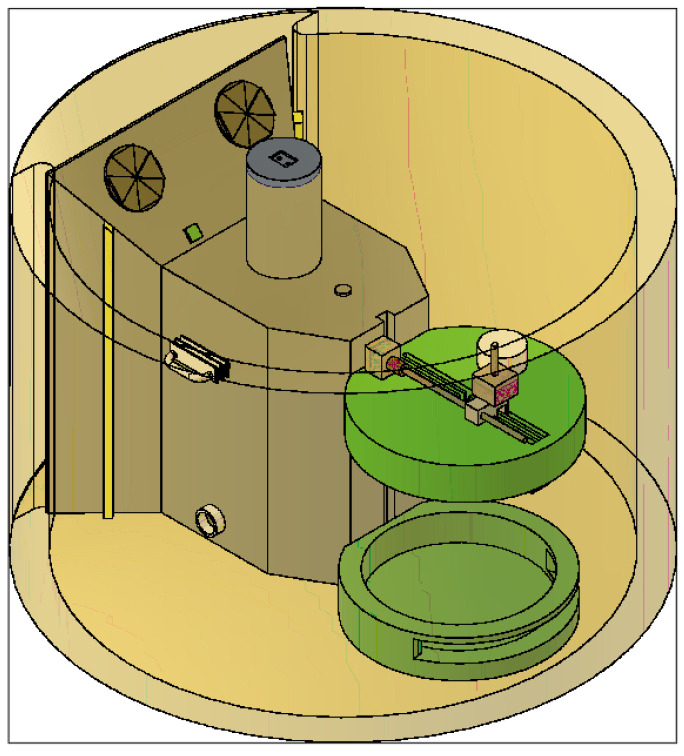
Test device for the optimisation of 3D bioprinting registered under patent number U202300008.

The hydrogels were introduced into vials to perform different printability tests using the test device (Figure 2, patent registered at the Spanish Patent and Trademark Office under number EN 1 303 662 U) [22]. This device was specifically designed to perform the basic tests to characterise the printability of hydrogels, including the grid test, the collapse test and the observation of the gelation state, while allowing the sterility conditions, the printing temperature and the humidity inside the device chamber to be controlled in case different tests involving the use of cells are desired.

Each hydrogel was left in the chamber for 5 min prior to testing to ensure proper stabilisation, and the humidity was adjusted to 95% to ensure maximum cell viability.

The temperature and pressure parameters applied to the hydrogel were calibrated according to the characteristics of the hydrogel to achieve maximum printability. Once the parameters were set (see Table 2), each test was replicated up to three times to ensure the reliability of the data. 

#### 2.3.5. Viability Assay

As shown in the study by Carpentier et al. [26], after performing an MTS assay, it was observed that GelMA showed adequate biocompatibility, making it a suitable compound for a cell viability assay. 

Each hydrogel (GelMA 100%, GelMA 75% and GelMA 50%), after knowing its rheological and printability properties and therefore the optimal printing pressure and temperature setting for each (as indicated in Table 2), was mixed with culture medium and a known concentration of HeLa cells and printed under controlled temperature and humidity conditions using the BIO X bioprinter. The required amount of each cell-loaded hydrogel was bioprinted to cover the bottom of each well of a 24-well multiwell cell culture plate, up to a total of 3 wells per hydrogel, and incubated for 24 h. Finally, the hydrogel was tested for viability using the LIVE/DEAD kit (Invitrogen, Waltham, MA, USA).

## 3. Results

Rotational rheology tests reproduce the behaviour of the material under shear stress, i.e., due to shear forces parallel to the plane of deformation of the material. These tests allow us to predict the response of the material during the bioprinting process and to relate this behaviour to its composition and internal structure since parameters such as viscosity, elastic modulus and viscoelastic behaviour influence parameters such as stiffness, flow through the nozzle or the ability of the hydrogel to maintain the structure generated after bioprinting [27]. Rheological tests were used to characterise the hydrogels, the results of which are discussed below.

### 3.1. Characterisation of the GelMA 100% Hydrogel

Rheological characterisation tests of the GelMA hydrogel were carried out to analyse the behaviour of the material and relate it to its composition and internal structure. Similar results to previous research have been observed in experimental analyses of the GelMA hydrogel in the absence of culture medium [28,29,30].

**Figure 3:**
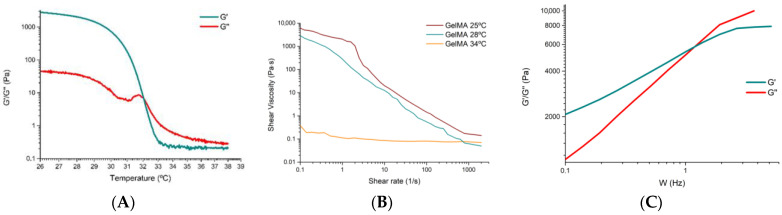
(**A**) Analysis of G′ and G″ at different temperatures for GelMA hydrogel. (**B**) Performance of GelMA hydrogel in the shear rate versus shear viscosity rheology test at 25 °C, at 28 °C and at 34 °C. (**C**) Oscillatory test of GelMA hydrogel to determine G′ and G″ at different frequencies at a set point temperature of 30 °C.

As no comprehensive rheological characterisation was available from the distributor to determine how temperature affected the internal structure of the hydrogel, the storage modulus (G′) and loss modulus (G″) were investigated. The elastic or storage modulus G′ represents the elastic component of the product and is directly proportional to the energy stored by the material in one deformation cycle. The viscous or loss modulus G′ represents the viscous behaviour of the sample and is directly proportional to the energy dissipated by the material in one deformation cycle. This test allows the rheological behaviour of the ink to be determined at different temperatures, and the results obtained are shown in Figure 3A. The test was performed between 26 °C and 39 °C so that the target temperature for bioprinting with cells (37 °C) would be within the range. 

In this figure, at 26 °C, the modulus G′ shows a higher value than the modulus G″, because the hydrogel shows a more elastic behaviour (gel state). At a temperature close to 32 °C, we observed the cut-off point between G′ and G″, so we can affirm that this point, which marks the gelation temperature, informs that at temperatures above 32 °C the hydrogel presents a fluid behaviour, less internal cohesion between the molecules, due to the fact that G″ is higher than G′, and that at temperatures below 32 °C the hydrogel manifests a gel state. The results are similar to those reported in the literature, with small deviations due to the experimental conditions (temperature, origin of the hydrogel or equipment used) [31,32].

Once the temperature at which the transition between the gel and viscous (fluid) states is determined, the shear behaviour versus shear rate at different temperatures of the hydrogel is analysed.

The response of the shear stress versus shear rate or shear rate of the GelMA for different temperatures (26 °C and 28 °C) is within the range where G′ is higher than G″ (in this range, the behaviour is predominantly elastic, so some fundamental mechanical properties for the bioprinting process would be improved). Also, the temperature of 34 °C is also investigated because it is close to the optimal temperature for maximum cell viability (37 °C).

Figure 3B shows the shear stress versus shear rate at 25 °C, 28 °C and 34 °C. A pseudoplastic behaviour is clearly observed at both 25 °C and 28 °C where the viscosity decreases with increasing shear stress or, in other words, the flow resistance decreases as the shear rate increases. It is observed that the viscosity varies as a function of temperature or applied shear stress. 

The GelMA hydrogel shows signs of shear thinning behaviour at 25 °C. Initially, there is a slight tendency for the apparent viscosity (a complex property that combines the dynamic viscosity of the pure fluid with the interactions that occur between the fluid and the solid surfaces or particles present in the medium) not to change in proportion with respect to the shear rate. This occurs between 0.1 s^−1^ and 2.51 s^−1^. From this interval, and with an apparent viscosity of approximately 3.15 × 10^2^ Pa·s, the power law zone begins, with the apparent viscosity of the hydrogel decreasing to 1.74 × 10^−1^ Pa·s. From this point, which coincides with the second Newtonian plateau, the apparent viscosity becomes constant for any shear rate.

At a temperature of 28 °C, the initial apparent viscosity decreases to a value of 2866 Pa·s, which is much lower than the initial apparent viscosity offered by the hydrogel at a temperature of 25 °C, where it was close to 6125 Pa·s. Moreover, at a temperature of 28 °C, the shear thinning behaviour is no longer so visible, although it still shows a decrease in viscosity that varies as a function of temperature or applied shear stress, which is typical of pseudoplastic materials.

Finally, at a temperature of 34 °C, the GelMA hydrogel shows a change to the point where it ceases to behave as a pseudoplastic material and exhibits a behaviour more typical of a Newtonian material, where the apparent viscosity becomes practically constant over the whole range studied. This result is related to the results obtained in the analysis of the storage and loss modulus, where the hydrogel behaves more like a liquid from 32 °C (Figure 3B), which makes it much less interesting and useful for bioprinting, among other reasons, because it will not be able to guarantee correct shape fidelity and printability nor sufficient mechanical properties to generate vertical structures.

From these results, it was concluded that the optimum temperature for bioprinting GelMA varies between 28 °C and 32 °C. At temperatures close to 28 °C, we found predominantly elastic behaviour, higher viscosity, shear thinning behaviour and better mechanical properties than at temperatures close to 32 °C, where the behaviour is more viscous and Newtonian. However, the temperature is optimal for achieving the highest possible cell viability.

Oscillatory tests, using a frequency sweep for deformation, allow the viscoelastic response to be monitored: shear storage modulus (G′) and loss modulus (G″) [30].

After the oscillatory test, the results obtained are shown in Figure 3C. This figure shows an interlocked type of behaviour (biopolymer) [33]. The first region where G′ is greater than G″ is the elastic region. The point or frequency where G′ and G″ intersect is the response time of the material and occurs at a frequency of 1.39 Hz. This point marks the end of a rubbery behaviour, where the material is more elastic, and the beginning of the viscous zone, where the material starts to flow, approaching a more liquid behaviour.

It can therefore be said that at low frequencies (<1.39 Hz) the hydrogel studied behaves like a gel, and at higher frequencies it behaves like a viscous material.

### 3.2. Rheological Characterisation of the Culture Medium and Buffers

In order to analyse how the culture medium affects the 3D bioprinting of the hydrogel, the shear behaviour versus shear rate of PBS and culture medium with and without FBS were analysed at temperatures between 25 °C and 37 °C.

According to Figure 3B, a temperature close to 28 °C offers high viscosity values for the GelMA 100% hydrogel, which could cause problems with the extrusion pressure of the hydrogel, and values above 32 °C could cause low viscosity and give poor quality prints.

Due to the above, the most interesting, expected temperature range for this study would be between 28 °C and 32 °C in terms of viscosity and rheological behaviour for 3D bioprinting of the GelMA hydrogel + culture medium.

**Figure 4:**
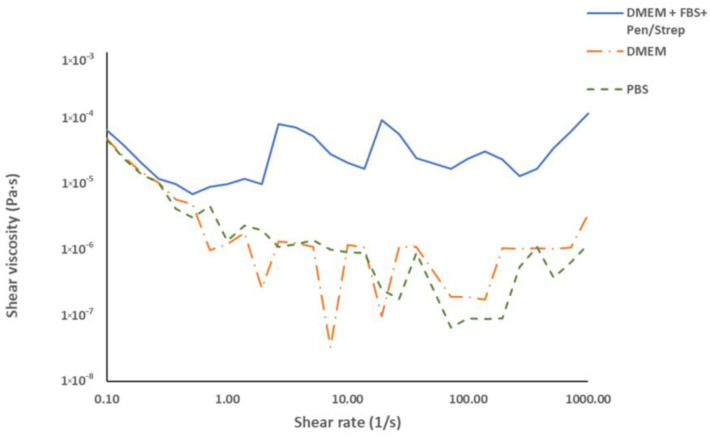
Behaviour of PBS, complete medium (DMEM + FBS + Pen/Strep) and medium without additives (DMEM) in the shear rate versus shear viscosity rheology test at 37 °C.

The results obtained are shown in Figure 4 for the temperature of 30 °C, although the behaviour obtained is very similar throughout the range studied. It was observed that, in all cases, the solutions exhibited a typical Newtonian behaviour, similar to that of water [34], where the values of shear stress and shear rate maintain a relationship of direct proportionality, which could influence the final behaviour of the hydrogel, that is, the apparent viscosity of the final hydrogel may decrease, directly affecting the printability, affecting the pseudoplastic behaviour and losing some very interesting properties.

It is important to note that the use of FBS in cell culture media raises ethical dilemmas and scientific challenges related to the variability in batch composition and quality, the risk of contamination with pathogens, its high cost and its incompatibility with clinical applications and production on a large scale [35].

### 3.3. Rheological Characterisation of GelMA Hydrogel with Culture Medium in Different Proportions

To analyse the influence of culture medium or buffer solutions on the GelMA hydrogel in 3D bioprinting, mixtures of GelMA and culture medium in different proportions were rheologically characterised. 

Figure 4 shows significant differences between the different solutions analysed, indicating that PBS and DMEM without FBS have similar rheological properties, while DMEM with FBS and Pen/Strep shows higher viscosity. These results indicate that DMEM with FBS and Pen/Strep, the most suitable medium for HeLA cell culture and one of the most widely used by the scientific community [36,37,38], is the compound with the highest viscosity and therefore the one that should least affect some properties of the formulated hydrogel, such as rheology or printability.

#### Analysis of the Storage Modulus as a Function of Temperature

**Figure 5:**
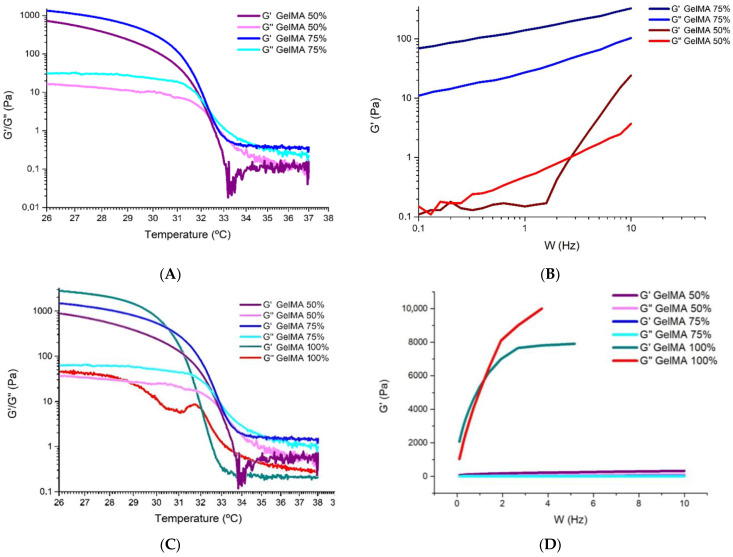
(**A**) Analysis of G′ and G″ at different temperatures for GelMA 75% and 50% hydrogel. (**B**) Oscillatory test to determine G′ and G″ at different frequencies for GelMA 75% and GelMA 50% hydrogel. (**C**) Analysis of G′ and G″ at different temperatures for GelMA 100%, 75% and 50% hydrogel. (**D**) Oscillatory test to determine G′ and G″ at different frequencies for GelMA 100%, 75% and GelMA 50% hydrogel.

Figure 5 shows the rheological analysis of the hydrogel GelMA 100%, GelMA 75% and GelMA 50%; in this way, we will analyse how the culture medium affects the rheological properties of the hydrogel.

In Figure 5A, we observe three zones for both the GelMA 75% hydrogel and the GelMA 50% hydrogel:

- Zone 1: at low temperatures (<32 °C), we see that G′ is higher than G″, so the ink is more elastic and approaches a gel state or behaviour.
- Zone 2: at around 32 °C, we observe that G′ intersects with G″, which marks the gelation temperature and indicates that the hydrogel gels have decreasing temperature.
- Zone 3: at temperatures above 32 °C, G′ equals G″ and is even slightly lower, again presenting a gel state behaviour.

In contrast to Figure 3A, which shows the behaviour of the 100% GelMA sample, Figure 5A shows that when culture medium is added and the temperature rises above 34 °C, G′ and G″ become equal, as the properties of the hydrogel become closer to those of a Newtonian material. It can also be seen that the apparent viscosity decreases over the whole range studied as the percentage of culture medium increases compared to 100% GelMA.

The ideal printing temperature range for the GelMA 75% and GelMA 50% hydrogels is between 30 °C and 32 °C (the zone where G′ is higher than G″, essential for high shape fidelity after extrusion) [20,30]. Although the temperature range is not ideal in terms of cell viability, it is within an acceptable range [20] where mechanical conditions would be maintained to ensure proper printability. 

The viscosity of both formulations decreased when combined with the culture medium, but the values were still within acceptable ranges [39]. This could solve extrusion problems caused by poor flow of the hydrogel inside the nozzle [40]. Therefore, based on the flowability of the hydrogel, the inclusion of different amounts of culture media on the final hydrogel could be very interesting.

The problem that GelMA 50% shows is a very low viscosity at 32 °C, so, in general, approaching temperatures close to 32 °C could present very inaccurate prints due to the hydrogel not having enough consistency, showing a very watery state.

The GelMA 75% hydrogel and the GelMA 50% hydrogel were subjected to an oscillatory test at the set point temperature of 30 °C. This temperature was selected because it ensures pseudoplastic and printability characteristics and is close to temperatures compatible with good cell viability values.

Figure 5B shows that the G′ and G″ curves of the GelMA 75% hydrogel do not intersect, indicating that the material has a gel-like behaviour at 30 °C. As there are no interlinks between the two curves shown, we can state that the hydrogel does not behave like a polymer [30].

At all times, G′ is greater than G″, so the hydrogel has a rubbery behaviour in which elastic characteristics predominate, i.e., the hydrogel has a behaviour that resembles that of a solid.

It can be observed that the GelMA 75% hydrogel has changed its behaviour with respect to the initial hydrogel (where G′ and G″ were cross-linked), having now a more elastic behaviour in the whole range studied.

In Figure 5B, the GelMA 50% hydrogel shows an interlocked type of behaviour (biopolymer). At low frequencies, G″ is higher than G′, which shows that the GelMA 50% has a viscous behaviour, i.e., the material flows. From a frequency of 3.16 Hz, G′ is higher than G″, giving way to an elastic behaviour in which the material shows a rubberier behaviour.

As demonstrated by the above and as shown in Figure 5C,D, the addition of culture medium causes a large variation in the behaviour of the hydrogel, but this does not mean that it is unsuitable in terms of printability.

### 3.4. Characterisation of Printability

For the printability studies, each of the analyses was repeated at least three times, selecting those tests with representative and reproducible results.

#### 3.4.1. Quantitative Assessment of the Gelation State and Printing Grid Test

This test aims to characterise extrusion hydrogels by performing two tests in one run. A filament with a good gel state is one that has a smooth surface and a constant width in all three dimensions, which facilitates the bioprinting of regular dies with square holes [22]. 

By analysing the gel state, we can obtain the printability (Pr), which is defined as follows [22,41]:

(1) $$Pr=\frac{\pi }{4}\cdot \frac{1}{C}$$

(2) $$If\;the\;figure\;is\;square\to C=\frac{4\pi }{16}\to Pr=\frac{\pi }{4}\cdot \frac{16}{4\pi }=1$$

**Figure 6:**
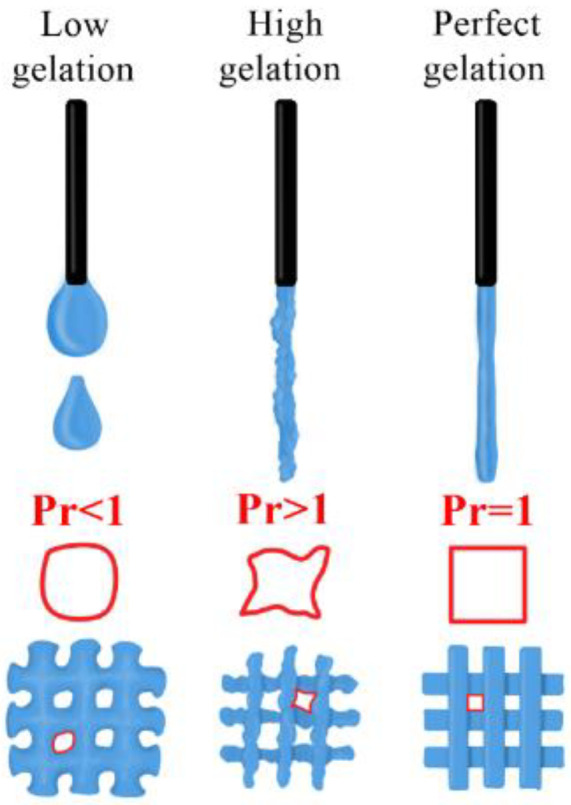
Comparative study of printability. Image from the INMA group. Image produced by the INMA group.

In addition, this test determines whether the hydrogel possesses sufficient mechanical properties to generate a “grid” or matrix formed with squares or rectangles of different dimensions (Figure 6). In other words, it tests the hydrogel’s ability to reproduce a given pattern and its tendency to generate accumulations or clusters.

To perform this test, the area in mm^2^ of the grid was first obtained/designed, which will be referred to as the theoretical value. Subsequently, the actual bioprinted area was measured, which will be referred to as the true value, and both values were compared using the statistical tool of standard deviation. In parallel, the value of Pr can be determined.

Knowing the dimensions of the designed matrix (theoretical value), the actual dimensions (real value) generated by the hydrogel when creating the matrix are obtained using an image processing programme.

Prior to carrying out the printability tests, the printing temperature of the hydrogels was calibrated within the range which, according to the rheological results, would show the best conditions for obtaining a good print, safeguarding the optimum conditions of cell viability as far as possible.

Therefore, the gelation of the hydrogels and their ease of extrusion were tested in the range between 28 °C and 32 °C. This range is defined by the predominance of pseudoplastic behaviour, and comprises the cut-off point between G′ and G″.

Both GelMA 75% and GelMA 50% showed very high levels of flowability at temperatures above 30 °C, so it was decided to continue the printability tests with a temperature value of 30 °C to try to get as close as possible to 37 °C (the temperature chosen for incubating the cells), obtaining an adequate level of gelation. In addition, to restore the structural capabilities of the hydrogels as quickly as possible, the temperature of the printing site was set at 20 °C, and UV light was applied with a 365 nm light-curing module at 3 cm from the bioprinted construct for 45 s.

**Figure 7:**
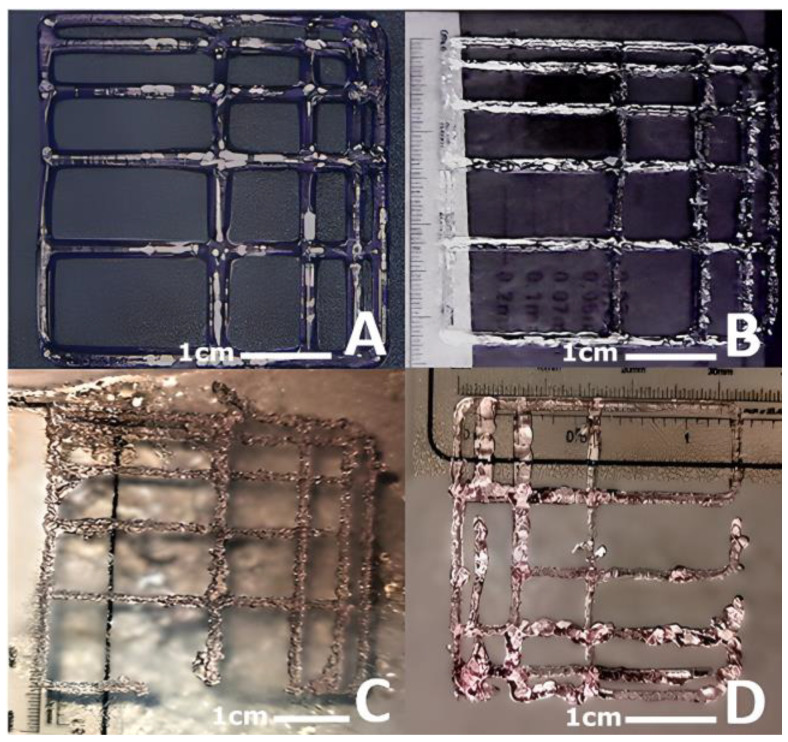
(**A**) CELLINK Start with Pr = 1 and appropriate gel state. (**B**) GelMA 100% with Pr > 1 and appropriate/high gel state. (**C**) GelMA 75% with Pr > 1 and appropriate/high gel state. (**D**) GelMA 50% with Pr > 1 and appropriate/high gelling state.

Figure 7 shows the results of the gelation state and printability of the GelMA 75% and GelMA 50% hydrogels. As a comparison, the GelMA hydrogels without culture medium and CELLINK Start have been added to analyse the differences in the level of printability observed, i.e., how the culture medium introduced influences the final hydrogel.

In the case of GelMA without culture medium, we see that it shows an adequate Pr. The hydrogel was able to print a square grid with angles close to 90 °C, although, because small hydrogel clots were visible, the gelation state was a bit high. 

The GelMA 75% and GelMA 50% hydrogels offer somewhat lower gelation values, mainly due to the introduction of culture medium, which makes the result more viscous overall. Both hydrogels studied showed an acceptable Pr and a gel state that allowed for complex prints at 30 °C to be considered.

The final printability results of the GelMA 50% indicate that, as its initial viscosity decreases and its elastic properties are lower, they are more influenced or conditioned by its homogeneity, so if the homogeneity is not adequate, the hydrogel will flow irregularly through the nozzle and the negative results will be more noticeable than with the GelMA 75%.

Figure 7 shows the printability results of the hydrogels studied: CELLINK Start, GelMA 100%, GelMA 75% and GelMA 50%. The real value, the theoretical value and the standard deviation of 20 grids were analysed for each hydrogel.

Table 3 and Table 4 show the values obtained for GelMA 75% and 50%. To calculate the deviation, the real value was subtracted from the theoretical value. For the creation of both tables, the parameters were adjusted (Table 1) and the test was repeated three times, collecting the most significant test values and averaging them. The smaller theoretical grid area (4 mm^2^) generated an overlap of layers, and correct bioprinting was not achieved in any of the cases.

Table 5 and Table 6 show the average deviation produced and the % similarity to the theoretical value for each hydrogel, within set ranges. In this way, it is easier to visualise which grid dimensions were most accurately reproduced by the hydrogels without significant overflows or printing faults.

For the realisation of both tables, three subgroupings of the printed grids were made for the realisation of Table 2 and Table 3 (theoretical grids with a value below 20 mm^2^, theoretical grids with a value between 20 mm^2^ and 64 mm^2^ and grids with a theoretical value equal to or greater than 64 mm^2^). For each subgrouping, the average deviation of each of the gratings and the final average percentage of similarity between the area of the theoretical grating and the final printed area were measured.

Table 5 shows that the GelMA 75% hydrogel shows greater similarity between the designed and subsequently bioprinted grid as the theoretical grid size increases. Thus, for grids with an area of less than 20 mm^2^, an accuracy level of 56.2% was achieved. This value, on the other hand, increases to 72.7% for grids with an area between 20 mm^2^ and 64 mm^2^, offering the maximum accuracy value for theoretical grids larger than 64 mm^2^, where an accuracy level of 91.28% was reached.

The GelMA 50% hydrogel shows, in the values shown in Table 6, a slight decrease in the similarity or similarity between the theoretical grid area and the real grid area, becoming more noticeable for low values of the theoretical grid area, where the percentage of similarity decreased to 51.77%. On the other hand, for a theoretical grid value between 20 and 40 mm^2^, the shape was reproduced with an accuracy level of 70.26%, only 2.44 points below the GelMA 75% hydrogel. For theoretical grid values above 64 mm^2^, the accuracy level increased to 87.91%, achieving much more promising results.

The difference between the theoretical value and the real value in the area measurements shown in Table 5 and Table 6 is due, in most cases, to the fact that the hydrogels did not achieve perfect 90° angles, as at some points several hydrogel layers overlapped, generating overflows that compromised the previously designed structure.

Table 7 shows the differences between the two hydrogels and the GelMA hydrogel without culture medium.

Table 7 shows that for theoretical grid values smaller than 64 mm^2^, GelMA with culture medium substantially worsens its printability properties, but for values equal to or larger (within the range studied) than 64 mm^2^, both hydrogels (GelMA 50% and GelMA 75%) offer very similar results in shape fidelity. This is why, to reproduce biomimetic structures of dimensions in the established range, the GelMA 50% hydrogel is very interesting, as it provides a larger amount of culture medium that improves the conditions of the cells.

#### 3.4.2. Strand Collapse Test

**Figure 8:**
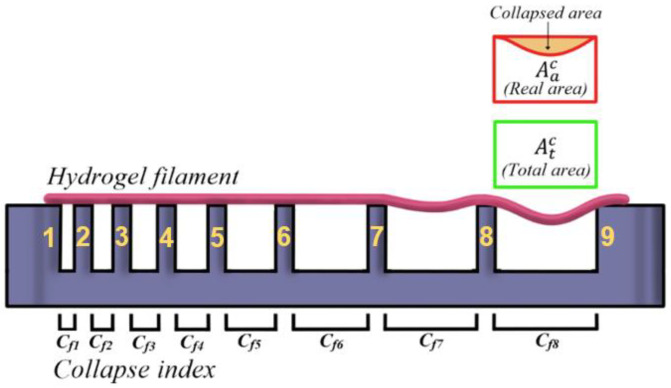
Measuring the collapsed area.

The objective of this test is to assess the ability of the hydrogel to form unsupported bridges. In other words, the aim is to determine the maximum distance the hydrogel can extend without collapsing (Figure 8).

**Figure 9:**
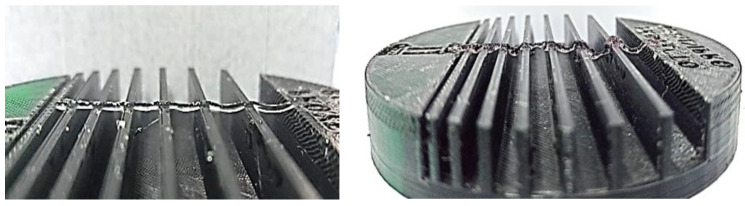
Collapse test. The image on the left shows the GelMA hydrogel without culture medium, and the image on the right shows the GelMA 50%.

To determine the collapsed area, a hydrogel filament was deposited on the pillars of a fixture (numbers 1 to 9 in Figure 8), and the deviation between the theoretical area and the real area was determined by measuring the area generated between the deposited hydrogel and each of the pillars [42]. In this way, the collapse index can be obtained (Figure 9):

(3) $$\;C_{f}=\frac{A_{t}^{c}-A_{a}^{c}}{A_{t}^{c}}\cdot 100\%\;$$

where $A_{t}^{c}$ is the total area, and $A_{a}^{c}$ is the area generated after depositing the filament.

If the filament manages not to collapse, coinciding with the real area and the total area, the collapse coefficient is 0%.

For the realisation of Table 8, the experiment was performed up to three times, and an average was obtained for each Cf value.

Table 8 again shows that, when the culture medium is introduced into the hydrogel, the structural capacity of the hydrogel is negatively affected, producing a greater collapse as the distance between pillars and the amount of culture medium introduced increases. It is observed that, while in GelMA 100% the collapse coefficient remains at zero until pillar number 6, in both GelMA 75% and GelMA 50% the collapse coefficient ceases to be zero at pillar number 3, producing a greater deflection for GelMA 50% at that point.

It should be noted that in no case did the collapse occur completely, so that both GelMA 50% and GelMA 75% presented acceptable deflection values and could generate complex biomimetic structures that needed to support unsupported impressions of up to 7.5 mm in length (maximum distance studied). 

### 3.5. Toxicological Characterisation

HeLa cells, a cervical adenocarcinoma cell line obtained from the Research Support Services of the University of Extremadura (SAIUEx), were used for toxicity testing. Hela cells were chosen for this study because they are a common model in cell biology and have contributed to many important discoveries, because they can adapt to different growth conditions and cell culture media, and because they are very easy to culture [43]

Cells were cultured in DMEM medium (Thermo Fisher) supplemented with 10% FBS (Gibco, Thermo Fisher, Waltham, MA, USA) and 1% penicillin/streptomycin (Sigma Aldrich) at 37 °C in a 5% CO_2_ incubator. The medium was changed each time acidification occurred due to the accumulation of acidic metabolites produced by the cells, and subcultures were prepared twice a week. Passage was performed with trypsin/EDTA solution (Sigma-Aldrich). Cells were counted in a haemocytometer to determine the total number of cells and then diluted to the desired concentration. 

Pure GelMA (GelMA 100%) and GelMA and culture medium in ratios of 1:3 (GelMA 75%) and 1:1 (GelMA 50%) were deposited as droplets on a 24-well multiwell cell culture plate by bioprinting using the Cellink BIO X bioprinter with a 22 G injector. All tests were performed in triplicate and measured after 24 h. 

**Figure 10:**
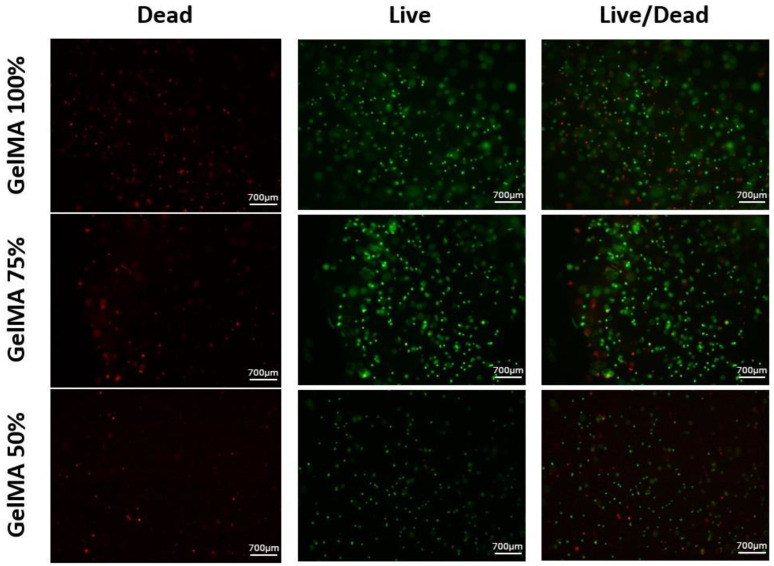
Viability test performed on GelMA 100%, GelMA 75% and GelMA 50% hydrogels.

Viability testing was performed at 24 h using the Invitrogen™ LIVE/DEAD assay to determine if there was a difference between adding more culture medium. Fluorescence images were taken using a Nikon Eclipse 80i fluorescence microscope (Figure 10). 

The images in Figure 10 show a high number of viable cells. Visually, there are no significant differences between the three types of hydrogels.

Cell viability was determined using Fiji digital processing software, which provided viability percentages for each hydrogel. The analysis resulted in 80.8% viable cells in the GelMA 50%, 77.3% in the GelMA 75% and 76.1% in the GelMA 100%.

## 4. Conclusions

The culture medium used in 3D bioprinting can affect the printability of hydrogels. This is because the culture medium has a low viscosity and a direct relationship with the shear stress, which can make it difficult to print complex structures. However, if the original hydrogel has a high viscosity, the culture medium can help to improve the creep of the material and therefore its printability.

In this manuscript, DMEM + FBS + Pen/Strep, DMEM and PBS were analysed, and the former was selected for the formulation of the hydrogels studied (GelMA 50% AND GelMA 75%), as it had a higher viscosity and should therefore have the least influence on the printability and rheological properties of the formulated hydrogels.

The GelMA hydrogel also exhibits pseudoplastic behaviour, with decreasing viscosity at higher strain rates, making it suitable for 3D bioprinting. The optimal printing temperature is around 30 °C, as lower temperatures hinder cell survival and higher temperatures compromise structural integrity. This behaviour is attributed to shear thinning, with two Newtonian plateaus and a power law region observed at 28 °C.

Hydrogels formed with 75% GelMA and 25% culture medium or 50% GelMA and 50% culture medium have similar pseudoplastic properties as the GelMA hydrogel without culture medium. However, the initial viscosity of the hydrogels with culture medium is significantly lower, especially for the hydrogel with 50% culture medium. The optimum printing temperature for both hydrogels is close to 32 °C, but the 50% medium hydrogel may not be viscous enough at this temperature, resulting in an inaccurate print.

Frequency oscillatory tests and analysis of the storage modulus as a function of temperature have shown a predominantly elastic behaviour for the hydrogels studied, especially at frequencies below 1.3 Hz or up to temperatures of 32 °C.

The GelMA hydrogel initially presented a gelation state suitable for bioprinting, obtaining a % similarity between the theoretical grid and the printed grid of more than 70% in all the areas studied and reaching a value of more than 90% for grids with an area greater than 64 mm^2^. Furthermore, in the collapse test, no considerable collapsed areas were observed in any case. Therefore, this hydrogel is very suitable in terms of printability.

The printability properties of GelMA 75% and GelMA 50% hydrogels were reduced compared to the GelMA hydrogel without culture medium. Both offer slightly lower gelation values, mainly due to the introduction of culture medium, which makes the overall result less viscous. Both hydrogels tested showed an acceptable Pr and a gel state that allowed complex prints at 30 °C and very consistent results in terms of shape fidelity.

When the culture medium is introduced into the hydrogel, the structural capacity of the hydrogel is negatively affected, with more collapse occurring as the distance between pillars and the amount of culture medium introduced increases. 

Both GelMA 50% and GelMA 75% hydrogels showed acceptable deflection values to support unsupported impressions up to 7.5 mm in length (the maximum distance studied). However, in the Cf7 and Cf8 tests, the GelMA 50% started to show a significant collapse which, depending on the structure to be printed, could be relevant to the final result, which was not as marked in the GelMA 75%. This suggests that GelMA 50% suffered a loss of mechanical capacity.

The toxicity test revealed a high number of viable cells with no significant differences between the two hydrogels (GelMA 50% and GelMA 75%), implying that the hydrogels were suitable for cell growth.

The results indicate that the GelMA 75% hydrogel is the best choice for bioprinting complex structures. While there are no significant differences in cell viability between the different hydrogels, the GelMA 75% hydrogel excels in the grid test and the collapse test. These two tests are important to assess the ability of the hydrogel to maintain its structure during and after bioprinting.

## Figures and Tables

**Table 1:** GelMA 10%/DMEM ratios.

Concentration		Proportion	Hydrogel Nomenclature
GelMA 10%	DMEM		
100%	0%	1:0	GelMA 100%
75%	25%	3:1	GelMA 75%
50%	50%	1:1	GelMA 50%

**Table 2:** Conditions applied to each of the hydrogels in the 3D bioprinting process. The UV model and UV exposure time were chosen because of previous results reported in other research [23,24,25].

Hydrogel	Printing Temperature (°C)	Pressure [kPa]	Injector Used	Impression Plate Temperature (°C)	Photoreticulation Module	Exposure Time to Ultraviolet Light (s)
Cellink Start	30	81	22 G	20	None	None
GelMA 100%	30	145	22 G	20	365 nm	45
GelMA 75%	30	138	22 G	20	365 nm	45
GelMA 50%	30	118	22 G	20	365 nm	45

**Table 3:** Grid test results for GelMA 75%.

	Real Value (mm^2^)	Theoretical Value (mm^2^)	Deviation (mm^2^)
Row 1	-	4.00	-
	4.29	8.00	3.71
	7.67	16.00	8.33
	11.52	32.00	20.48
Row 2	6.88	8.00	1.12
	12.54	16.00	3.46
	25.50	32.00	6.50
	57.35	64.00	6.65
Row 3	6.30	12.00	5.70
	19.19	24.00	4.81
	42.10	48.00	5.90
	92.24	96.00	3.76
Row 4	12.00	16.00	4.00
	27.32	32.00	4.68
	56.70	64.00	7.30
	119.32	128.00	8.68
Row 5	11.70	20.00	8.30
	32.67	40.00	7.33
	67.14	80.00	12.86
	154.00	160.00	6.00

**Table 4:** Grid test results for GelMA 50% hydrogel.

	Real Value (mm^2^)	Theoretical Value (mm^2^)	Deviation (mm^2^)
Row 1	-	4.00	-
	4.20	8.00	3.80
	13.95	16.00	2.05
	14.50	32.00	17.50
Row 2	4.70	8.00	3.30
	7.50	16.00	8.50
	25.78	32.00	6.22
	51.20	64.00	12.80
Row 3	6.10	12.00	5.90
	19.92	24.00	4.08
	39.68	48.00	8.32
	87.78	96.00	8.22
Row 4	10.60	16.00	5.40
	24.80	32.00	7.20
	52.10	64.00	11.90
	124.20	128.00	3.80
Row 5	12.00	20.00	8.00
	25.12	40.00	14.88
	69.24	80.00	10.76
	145.64	160.00	14.36

**Table 5:** Percentage comparison shown by the GelMA 75% hydrogel between theoretical grid area and bioprinted grid area.

	Number of Samples	Average Deviation Produced (mm^2^)	% Similarity to Theoretical Value
Theoretical value x < 20 mm^2^	7.00	4.39	56.20
Theoretical value 20 mm^2^ ≤ x < 64 mm^2^	7.00	8.29	72.70
Theoretical value ≥ 64 mm^2^	6.00	7.54	91.28

**Table 6:** Percentage comparison shown by the GelMA 50% hydrogel between theoretical grid area and bioprinted grid area.

	Number of Samples	Average Deviation Produced (mm^2^)	% Similarity to Theoretical Value
Theoretical value x < 20	7.00	4.83	51.77
Theoretical value 20 ≤ x < 64	7.00	9.46	70.26
Theoretical value ≥ 64	6.00	10.31	87.91

**Table 7:** Comparison of the % similarity between the theoretical grid and the bioprinted grid with each hydrogel.

	GelMA 75%	GelMA 50%	GelMA
Theoretical value x < 20	56.20	51.77	76.76
Theoretical value 20 ≤ x < 64	72.70	70.26	88.58
Theoretical value ≥ 64	91.28	87.91	91.85

**Table 8:** Collapse test performed on different hydrogels. Values are given in percent.

Hydrogel	*C_f_*_1_	*C_f_*_2_	*C_f_*_3_	*C_f_*_4_	*C_f_*_5_	*C_f_*_6_	*C_f_*_7_	*C_f_*_8_
GelMA 100%	0.00	0.00	0.00	0.00	0.00	0.85	0.95	1.10
GelMA 75%	0.00	0.00	1.52	2.23	3.23	6.48	8.48	8.20
GelMA 50%	0.00	0.00	2.27	3.03	7.86	10.67	14.70	15.10

## Data Availability

Data are contained within the article.
